# Pneumothorax spontané: mode de révélation inhabituel d’un hamartochondrome pulmonaire

**DOI:** 10.11604/pamj.2016.24.289.10186

**Published:** 2016-07-29

**Authors:** Hind Serhane, Oussama Abdessalam Afandi, Yassine Msougar, Lamyae Amro

**Affiliations:** 1 Service de Pneumologie, Hôpital Arrazi, CHU Mohammed VI, Marrakech, Maroc; 2 Service de Chirurgie Thoracique, Hôpital Arrazi, CHU Mohammed VI, Marrakech, Maroc

**Keywords:** Hamartochondrome pulmonaire, hamartome pulmonaire, pneumothorax spontané, tumeur bénigne du poumon, formations kystiques, épanchements pleuraux, Pulmonary hamartoma, spontaneous pneumothorax, benign lung tumor, cystic formations, pleural effusions

## Abstract

L'hamartochondrome est une tumeur bénigne de l'arbre trachéo-bronchique de découverte fortuite le plus souvent et rarement symptomatique. Elle est plus fréquente chez l'homme que chez la femme. L'aspect radiologique est souvent évocateur. Le recours à la chirurgie est indiquée quand la tumeur est de grande taille et/ou symptomatique. Le diagnostic histologique ne pose en règle pas de difficulté. Nous rapportons l'observation d'une jeune patiente de 30 ans, sans antécédents pathologiques particuliers. Qui avait présenté un pneumothorax spontané, révélant un hamartochondrome pulmonaire de grande de taille. Association assez rare, mais pourrait être expliqué par le fait que ces tumeurs de composition assez anarchique, contiennent parfois des formations kystiques qui peuvent se rompre dans la plèvre et être à l'origine d'épanchements.

## Abstract (en)

Hamartochondroma is a benign tumor of the tracheobronchial tree, often found incidentally and rarely symptomatic. It is more common in men than in women. Its radiological aspect is often evocative. Surgery is indicated for large and/or symptomatic tumors. Histological diagnosis usually poses no difficulty We report the case of a 30-year-old young patient with no particular past medical history and with spontaneous pneumothorax revealing a large sized hamartochondroma. This rare association could be explained by the fact that tumors of fairly anarchic composition may contain cyst formations that may rupture into the pleura causing effusions.

## Introduction

Le pneumothorax spontané est un mode de révélation des hamatochondromes pulmonaires, rarement rapporté dans la littérature [1]. Ces derniers sont des tumeurs bénignes, composées de façon anormale de composés tissulaires normaux de l'organe dans lequel ils sont trouvés, dans ce cas c'est le parenchyme pulmonaire. Les hamatochondromes pulmonaires contiennent du tissu cartilagineux souligné par de l'épithélium bronchique et du stroma fibromyxoïde. Ils peuvent également contenir de la graisse ou des collections kystiques liquidiennes [2]. Ils sont plus fréquents chez l'homme (surtout de plus de 60 ans) que chez la femme, avec un sexe ratio de 2,5. Le mécanisme par lequel cette complication survient n'est pas clair. Nous rapportons l'observation d'une jeune patiente présentant un harmatochondrome pulmonaire révélé par un pneumothorax spontané.

## Patient et observation

**Figure 1:**
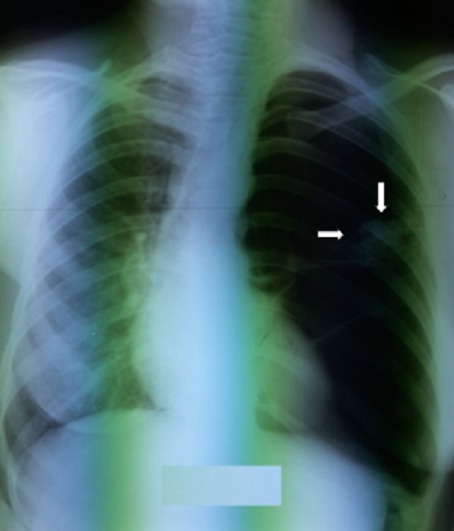
Radiographie thoracique à l’admission montrant le pneumothorax gauche avec l’opacité ronde

**Figure 2:**
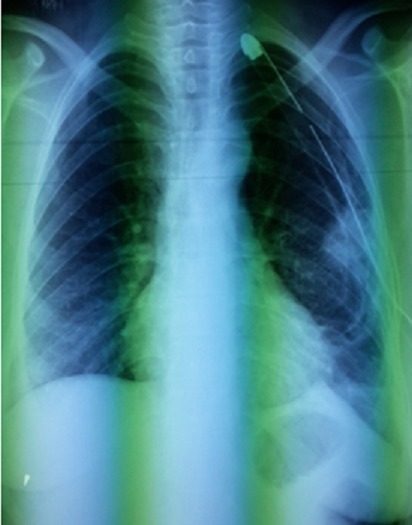
Radiographie thoracique après drainage

**Figure 3:**
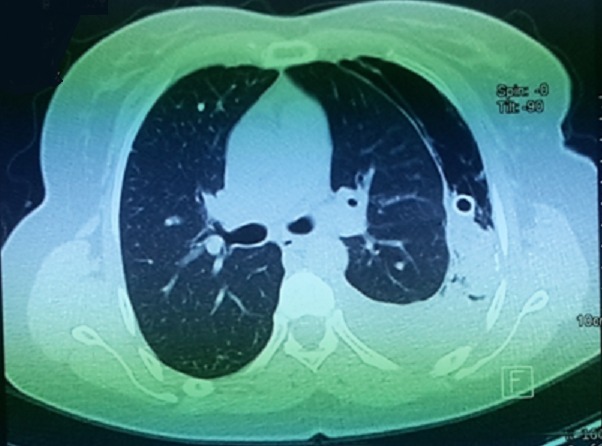
Aspect TDM

**Figure 4:**
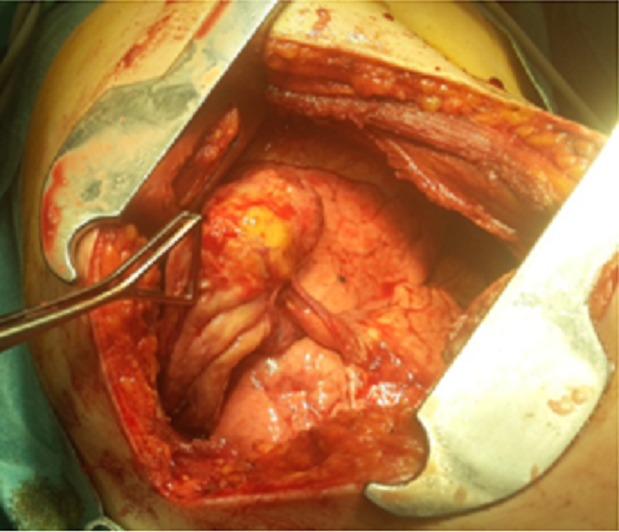
Vue per-opératoire de la masse tumorale

**Figure 5:**
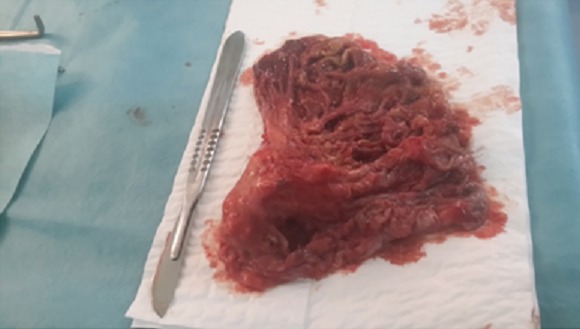
Aspect macroscopique de la tumeur après exérèse

Mme S.A âgée de 30 ans, non tabagique, sans antécédents pathologiques particuliers notamment pas de traumatisme thoracique ni contage tuberculeux récent. Elle avait présenté une douleur thoracique d’installation aigue, en coup de poignard, sans facteur déclenchant apparent. Cette douleur était associée à une dyspnée d'effort stade de II de Sadoul et une toux sèche. La patiente était en bon état général, l'envergure sur la taille était à 0,9 sans signes d'hyperlaxité ligamentaire. L'examen pleuro-pulmonaire avait objectivé un syndrome d'épanchement aérien occupant la totalité de l'hémithorax gauche. Le diagnostic de pneumothorax a été confirmé par la radiographie thoracique de face qui avait objectivé une hyperclarté sans trame vasculaire visible occupant la totalité de l'hémithorax gauche avec une opacité ronde, bien limitée mesurant 11cm dans son grand axe, homogène siégeant en axillaire gauche semblant se raccorder à angles aigus avec la paroi thoracique (Figure 1). Un drainage thoracique en axillaire gauche a été réalisé chez la patiente. Le cliché thoracique après drainage montre un retour du poumon à la paroi avec persistance de l'opacité ronde (Figure 2). Au 2ème jour du drainage, l'évolution s'est marquée par l'apparition d'une image hydro-aérique occupant la totalité de l'hemithorax gauche en rapport avec un hydro-pneumothorax, avec résolution sous aspiration continue. Un bilan étiologique a été réalisé: l'hémogramme, la CRP, l'ionogramme sanguin étaient normaux, l'intradermo-réaction à la tuberculine (IDR à t) était négative à 0 mm, 3 recherches de bacille de Koch (BK) dans les expectorations étaient négatives, la sérologie hydatique était négative, le taux de Ca 125 était normal à 27 UI/ml. La ponction pleurale avait montré un liquide exsudatif, jaune citrin à prédominance lymphocytaire. La bronchoscopie avait noté un état inflammatoire diffus de 1er degré sans bourgeon ni granulome visible, la recherche de BK dans le liquide d'aspiration bronchique s'était révélée négative, la culture de BK dans le liquide bronchique également. La TDM thoracique avait objectivé une opacité de densité liquidienne basale gauche surmonté d'une hyperclarté au sein de laquelle existait une image tissulaire bien arrondie difficilement individualisable sur les coupes parenchymateuses mesurant à peu près 10 cm dans son grand axe (Figure 3). Devant ce nodule de grand diamètre et dont l'origine n'a pu être déterminée, une exérèse complète a été réalisée par thoracotomie postéro-latéral gauche (Figure 4, Figure 5). L'étude de la pièce opératoire avait noté une masse blanc nacré, de consistance ferme et bien limitée, pesant 60g et mesurant 10 x 9 x2,5 cm. l'examen microscopique montre une prolifération carcinomateuse bénigne faite de chondroblastes, de chondrocytes réguliers, de lobules graisseux et de tissu cartilagineux bordé d'une muqueuse de type respiratoire régulière réalisant un aspect compatible avec un hamartochondrome pulmonaire. Les suites post opératoires étaient simples.

## Discussion

Devant un pneumothorax spontané chez une jeune femme ayant une masse intraparenchymateuse pulmonaire sur la TDM thoracique, certains diagnostics sont à évoquer de principe. Dans notre observation, l'origine infectieuse (tuberculose ou kyste hydatique rompu), ont été éliminés devant le contexte et le bilan infectieux (la recherche de BK, la sérologie hydatique, les prélèvements au cours de la bronchoscopie) qui étaient négatifs. Un pneumothorax cataménial sur endométriose thoracique a été éliminé aussi, vu le caractère non récidivent, la survenue à distance des menstruations et le taux de Ca 125 qui était normal. L'absence de lésions kystiques visibles sur la TDM thoracique permet d'éloigner une lymphangioléiomyomatose. Un pneumothorax d'origine métastatique était donc probable. L'état général conservé et l'absence de notion de tabagisme et de signes extra-respiratoires étaient en faveur de la bénignité de la lésion tissulaire. Un pneumothorax associé à une tumeur bénigne type hamartochondrome était un diagnostic plausible devant le contexte clinique et l'aspect scannographique. L'hamartochondrme (HC) mésenchymateux est la tumeur pulmonaire bénigne la plus fréquente (77% des tumeurs bénignes pulmonaires) [3]. Il s'agit d'une tumeur dérivée du tissu mésenchymateux péribronchique, constituée dans un désordre absolu et en proportions variables de cartilage, de tissu conjonctif, de graisse, de muscle lisse et d'épithélium respiratoire [4]. On distingue deux formes différentes par leurs manifestations cliniques que par leurs traitements et leurs aspects histologiques : l'HC intraparenchymateux (HCI) et l'HC extraparenchymateux (HCE). Les deux formes touchent préférentiellement l'adulte vers la soixantaine, de sexe masculin et tabagique [5]. La sex-ratio M\F est de 2,5 [6]. La découverte de l'hamartochondrome est le plus souvent fortuite et les circonstances de découverte sont non spécifiques [7]; toux sèche (11%), douleurs thoraciques (10%), expectorations mucopurulentes (5%), pneumopathies aiguës ou récidivantes (5%), dyspnée (5%), asthénie ou amaigrissement (4%), signes digestifs (2%) [6]. Dans notre observation, le pneumothorax spontané chez une femme jeune était un mode de révélation, inhabituelle et rarement rapportée dans la littérature. Un seul cas similaire était observé par Dian-bo Cao et al [1]. Comme dans l'observation rapportée, l'aspect radiologique de l'hamartochondrome est celui d'une opacité bien limitée et arrondie, parfois ovalaire ou lobulée. La présence de foyer de densité graisseuse ou de calcifications intratumorales « en pop-corn » ou en « grenaille de plomb » sur le scanner est évocatrice mais n'est pas systématique [6]. Dans notre cas, la tomodensitométrie ne montre ni calcification, ni plage de densité graisseuse. L'HC pulmonaire est le plus souvent de petite taille (entre 20 et 40 mm dans 50% des cas) et de localisation périphérique [6]. Chez notre patiente, il était de grande taille mais de localisation périphérique. La difficulté diagnostique réside dans le fait que le radiologue a parfois du mal à différencier entre le caractère bénin et malin de la lésion [8], comme dans le cas de notre patiente où le recours à la chirurgie était la solution pour trancher. Quand le caractère bénin de la lésion est affirmé, ou quand la lésion est de petite taille et sans expression clinique, la seule surveillance est suffisante. La chirurgie est donc indiquée quand la lésion est d'emblée de grande taille, comme le cas de notre patiente, quand au cours de la surveillance augmente de taille ou quand elle devient symptomatique [8]. Comme dans notre observation, le diagnostic anatomopathologique ne pose en règle générale pas de difficulté. Il s'agit d'un nodule bien circonscrit, de taille variable, lobulé, de coloration blanc grisâtre, légèrement brillant et de consistance ferme (Figure 5). Au plan histologique, les hamartomes pulmonaires sont composés en proportion variable de tissu cartilagineux souvent majoritaire, de tissu conjonctif et de plages de tissu adipeux [9]. En périphérie, ils sont entourés d'une assise de cellules épithéliales cylindriques, dont certaines sont ciliées ou mucipares.

## Conclusion

L'hamartochondrome pulmonaire est une tumeur bégnine de découverte souvent fortuite sur cliché thoracique ou devant des symptômes respiratoires non spécifiques, le pneumothorax est un mode de révélation inhabituel des hamartochondromes. Ceci probablement est expliqué par le fait que l'HC peut être constitué de lésions kystiques, de localisation périphérique proche de la plèvre. Ces lésions kystiques peuvent se rompre et être à l'origine d'épanchements. Ou, il s'agit tout simplement d'une association fortuite.
